# Genomics and proteomics in liver fibrosis and cirrhosis

**DOI:** 10.1186/1755-1536-5-1

**Published:** 2012-01-03

**Authors:** Rebekka A Hannivoort, Virginia Hernandez-Gea, Scott L Friedman

**Affiliations:** 1 Department of Medicine/Division of Liver Diseases, Mount Sinai School of Medicine, New York, NY 10029, USA; 2 Department of Gastroenterology and Hepatology, University Medical Center Groningen, University of Groningen, Groningen, The Netherlands

**Keywords:** cirrhosis, genomics, liver fibrosis, mass spectrometry, microarray, proteomics

## Abstract

Genomics and proteomics have become increasingly important in biomedical science in the past decade, as they provide an opportunity for hypothesis-free experiments that can yield major insights not previously foreseen when scientific and clinical questions are based only on hypothesis-driven approaches. Use of these tools, therefore, opens new avenues for uncovering physiological and pathological pathways. Liver fibrosis is a complex disease provoked by a range of chronic injuries to the liver, among which are viral hepatitis, (non-) alcoholic steatohepatitis and autoimmune disorders. Some chronic liver patients will never develop fibrosis or cirrhosis, whereas others rapidly progress towards cirrhosis in a few years. This variety can be caused by disease-related factors (for example, viral genotype) or host-factors (genetic/epigenetic). It is vital to establish accurate tools to identify those patients at highest risk for disease severity or progression in order to determine who are in need of immediate therapies. Moreover, there is an urgent imperative to identify non-invasive markers that can accurately distinguish mild and intermediate stages of fibrosis. Ideally, biomarkers can be used to predict disease progression and treatment response, but these studies will take many years due to the requirement for lengthy follow-up periods to assess outcomes. Current genomic and proteomic research provides many candidate biomarkers, but independent validation of these biomarkers is lacking, and reproducibility is still a key concern. Thus, great opportunities and challenges lie ahead in the field of genomics and proteomics, which, if successful, could transform the diagnosis and treatment of chronic fibrosing liver diseases.

## Introduction

Liver fibrosis results from a wound-healing response to chronic injury, which leads to excessive matrix, or scar deposition. This scar tissue can restrict blood flow due to contraction of the organ, leading to progressive liver damage and cirrhosis (the end stage of fibrosis), complicated by liver failure, portal hypertension and/or hepatocellular carcinoma [1]. Fibrosis is prominent in chronic liver diseases, including viral hepatitis, alcoholic and non-alcoholic steatohepatitis, toxic liver injury, auto-immune diseases and several genetic diseases. There have been two major priorities for therapy to reduce fibrosis: 1) to establish treatments for the diseases that lead to liver fibrosis; and, 2) to identify agents that directly slow or reverse fibrogenesis independent of the underlying disease.

A key discovery in understanding fibrosis has been the role of hepatic stellate cells (HSCs), vitamin A storing cells in the space of Disse, which, when activated, transform into myofibroblast-like cells, shedding their vitamin A content, and producing fibrogenic proteins, including collagens and tissue inhibitor of metalloproteinases-1 (TIMP-1) [2]. This review will focus on the contribution of high-throughput genomic and proteomic approaches to the study of fibrogenesis and fibrosis progression, concentrating on the most prevalent human chronic liver diseases and findings from animal models in liver tissue, isolated liver cells, cell lines and serum.

### The role of genomics and proteomics in degenerative diseases and liver fibrosis

Genetic diseases can be classified as chromosomal abnormalities (for example, trisomy 21), Mendelian disorders (single gene alterations with typical inheritance patterns, like autosomal dominant/recessive or X-linked), and complex diseases that are influenced by many genetic and environmental components. Degenerative diseases like liver fibrosis are complex illnesses [3]. The genetic contributions to these disorders are not attributable to a single gene alteration, but rather to a host of genetic susceptibilities defined by single nucleotide polymorphisms (SNPs) that predispose an individual to a disease. The susceptibility to an accumulation of environmental influences is either enhanced or reduced by genetic factors, thereby defining an individual's disease risk. Studies investigating these genetic traits are complicated, because there are many genes that influence the risk for complex diseases, yet the impact of each single genetic variant by itself is small. Therefore, large numbers of subjects are needed to provide sufficient statistical power to yield robust conclusions. Currently, there are almost 13 million SNPs catalogued in the NCBI human SNP database. Approaches to identify SNPs that are linked with a specific disease range from efforts to sequence specific disease-causing genes to genome scans requiring sequencing of large numbers of known SNPs that may or may not be associated with the disease.

Genomic and proteomic screening methods are often used to identify classes of genes that are differentially expressed in disease. These classes provide the investigator with potential pathways that could be involved in the regulation of this disease, thereby narrowing the focus of subsequent studies to uncover disease mechanisms and potential targets for therapy. By not limiting the study to pathways already associated with a disease, many new pathways not previously implicated are now under investigation [4].

This prospect is especially compelling in degenerative diseases like liver fibrosis, as these conditions often develop slowly, and therapies may not be necessary for all subclasses of patients [5]. Regardless of the etiology of liver fibrosis, some people progress rapidly towards cirrhosis, whereas others never develop fibrosis in the first place, or have slow progression of their fibrosis. It seems very unlikely that this phenomenon can be attributed solely to environmental influences. Identifying which patients are unlikely to ever progress to cirrhosis may prevent overtreatment of many patients.

Another use for these screening methods is to identify expression profiles, or patterns of expression of many genes, that in aggregate correspond with disease outcome or the response to therapy. By analyzing these different gene/proteome classes, investigators may ultimately predict an individual patient's response to therapy more accurately, or identify those who are at greatest risk of progression, thereby refining treatment decisions.

### The need for non-invasive markers in the assessment of fibrosis progression

The need to assess hepatic fibrosis progression is becoming more important as the incidence of advanced liver disease continues to rise, especially due to viral hepatitis and fatty liver disease. Liver biopsies are currently the 'gold standard' for determining the presence and progression of liver fibrosis. They are performed percutaneously, via transjugular access, or during abdominal surgery. Liver biopsies have a low complication rate; however, significant hemorrhage may rarely occur, requiring hospitalization in approximately 2% of the patients; moreover, some patients have contraindications for liver biopsy [6-8]. Additionally, analysis of liver biopsy is often undermined by sampling error and inter-observer variation [ [6,9-15]. Studying mRNA and protein disease profiles in liver biopsies is challenging, as tissue sample sizes are small, especially for protein analysis, and the biopsies contain a mix of many different cell types, including hepatocytes, hepatic stellate cells, Kupffer cells, endothelial cells, lymphocytes, red blood cells and bile duct epithelial cells. To study a single cell population from tissue sections requires laser-capture micro-dissection, which yields even less mRNA and protein than biopsy [15].

Close follow-up of liver disease by serial biopsy is not practical due to its invasive nature, fueling the need to find alternatives for liver biopsy. A less invasive and more accurate diagnostic tool would aid in diagnosis of fibrosis, follow-up of established therapies and evaluation of experimental therapies. Moreover, hepatocellular carcinoma (HCC) is one of the main complications of cirrhosis, and early detection markers that distinguish HCC from cirrhosis could significantly reduce morbidity and mortality. In the past ten years, genomic, transcriptomic and proteomic studies have provided candidate biomarkers for fibrosis assessment. Thus, in combination with standard laboratory serum analysis, these have led to novel fibrosis assessment tools using indirect and direct fibrosis markers, like FibroSpect (Prometheus Laboratories; San Diego, CA, US), FibroTest (BioPredictive;Paris, FR) and Fibroscan (Echosens; Paris, FR), ActiTest (BioPredictive;Paris, FR) and APRI (public domain) [16-20]. For a more exhaustive overview of currently used serum biomarkers, see a recent review by Smith *et al*. [21].

These fibrosis assessment tests have shown promising results, but a number of pitfalls are encountered, including limited availability, inter-laboratory variation, cost, insufficient resolution to differentiate between intermediate stages of fibrosis and false positive results due to other conditions, such as increased venous pressure or steatosis [22-29].

Because current methods for non-invasive assessment of liver fibrosis lack resolution, particularly in the intermediate fibrosis stages, more research is being directed towards genomics and proteomics in the search of biomarkers that provide a close association with fibrosis stage [30-35].

## Technical aspects of genomics and proteomics in chronic liver disease

### Genomics and transcriptomics

Genomics comprises the study of the genetic information (DNA, or RNA in certain viruses) of an organism. Transcriptomics aims to elucidate the transcripts of the genome or gene expression levels on the RNA level under varying conditions as changes in mRNA expression do not necessarily correspond with changes in protein expression, since alternative splicing, protein production, degradation and post-translational modifications influence protein stability and function [36,37].

The cDNA microarray is the most frequently used high-throughput screening method for gene expression profiling. These 'lab-on-a-chip' assays contain specified DNA oligonucleotide probes in spots on a chip that hybridize with oligonucleotides from tissue or cells providing a quantitative assessment of the transcribed genes [42-44]. Thousands of genes can be analyzed simultaneously with cDNA microarray and variants on this technique include specialized arrays that can detect SNPs and alternative splice variants (exon junction arrays) [38-41].

Serial analysis of gene expression (SAGE) analyzes the transcriptome by cloning strings of short cDNA fragments into bacteria, sequencing the cDNA fragments and then counting the number of cDNA fragments. This technique can yield information on both gene expression and alternative splicing and can help identify previously unknown genes [42,43].

Target genes that are revealed by the genomics/transcriptomics screens are generally validated by reverse transcription quantitative polymerase chain reaction (RT-qPCR), which is more quantitative and more reproducible than microarray, requires less mRNA and is more sensitive for genes that are expressed at low levels.

### Proteomics

Proteomics is the study of the proteome in a cell compartment, tissue or organism comprising all proteins that are encoded for in the genome. Mass spectrometry (MS) is the key technique in proteomics. Many variants of mass spectrometers are currently in use, but they all rely on the same concept: to determine the accurate mass of a protein by measuring the mass-to-charge (*m/z*) ratio. In short, mass spectrometers consist of an ion source, a mass analyzer and a mass detector. After ionization, proteins and peptides travel through the mass analyzer, which evaluates their ratio of charge (*z*) versus mass (*m*). The mass detector counts the number of molecules per charge-to-mass ratio, and can provide the user with an output as a mass spectrum, with the *m/z *ratio on the x-axis and the molecule count per *m/z *on the y-axis [44].

MS is preceded by a separation step of proteins, either through 2D-polyacrylamide gel electrophoresis (2-DE/2D-PAGE), liquid chromatography (LC) or gel electrophoresis (DIGE) [45-47]. Once the protein spots are excised from the gel and digested, they can be processed through one mass analyzer (MS) to identify peptide mass, or two mass analyzers (MS/MS) to determine amino acid sequence.

MS requires ionization of proteins and peptides and two soft-ionizing techniques have been developed: matrix-assisted laser desorption/ionization (MALDI) and Electrospray ionization (ESI). Both techniques charge molecules that are subsequently analyzed by one or two mass analyzers (MS or versus tandem MS/MS). Time-of-flight (TOF) mass analyzers deduct peptide mass by measuring the time it takes for a charged peptide to travel through a vacuum tube in an electric field and can also be combined with one (Q-TOF) or two quadrupoles (Q-Q-TOF), which uses an oscillating electric field to selectively allow for molecules with a specific range of *m/z *ratios to proceed without collision [48].

When extremely high resolution is required, Fourier transform-ion cyclotron resonance (FT-ICR) MS or orbitrap can be used. The charged molecules are injected into a Penning ion trap where detectors measure the signal of molecules that pass. The *m/z *ratio determines the frequency with which the ion passes over the detector [49-51].

Surface-enhanced laser desorption/ionization (SELDI) analyzes protein mixtures that selectively bind to a biochip with the characteristics of choice. It needs only a small amount of crude sample (for example, serum or small liver biopsies), and is suitable for profiling multiple low-molecular-weight proteins. SELDI does not provide direct identification of the proteins. However, the peak profiles generated by SELDI can be useful by themselves in diagnostics, or in predicting prognosis and/or treatment response. Since manual labor is minimal, SELDI is by itself a suitable technique for high-throughput screening, especially for serum biomarkers; however, the cost is still too high for application on a large clinical scale [52-54]. For an excellent overview of the different types of mass spectrometers and their individual strengths and weaknesses, see a review by Domon and Aebersold [55].

A different type of high-throughput screening of proteins is the protein microarray. It functions much like a cDNA microarray, but probes are constructed out of proteins, antibodies or DNA constructs containing protein binding sequences that can capture proteins on a chip [56].

### Technical pitfalls

Technical variations are largely responsible for the low reproducibility between studies by different research groups. Differences in patient characteristics can significantly alter genomic and proteomic profiles. Proteome studies are especially difficult because proteins cannot be amplified, unlike DNA and mRNA. Therefore, scientists have to rely on fractionation, enrichment/depletion and solubilization protocols prior to MS to prevent overload with the most abundant proteins present in the serum urine

## Overview of genomics and proteomics in chronic liver disease

Most chronic liver diseases lead to fibrosis and cirrhosis in a significant subset of patients. While many common pathways drive fibrogenesis in all these diseases, there are also disease-specific pathways that contribute to fibrosis. An increasing number of studies are comparing the transcriptome and proteome of patients with different types of chronic liver injury to unearth disease-specific abnormalities in gene or protein expression.

In the following section we focus on disease-specific genomic and proteomic studies in both animal models and human diseases.

### In vivo *and *in vitro *animal models of fibrosis*

Not all human liver diseases can be reproduced accurately in animal models, and thus surrogate models are being developed to study mechanisms of chronic liver injury and fibrosis.

Rodent studies reviewed below have analyzed a variety of fibrosis progression and resolution models, and have evaluated liver tissue, cell isolates, serum and urine proteomes. Considerable additional effort will be required to systematically compare proteomes between different disease models, and to accomplish the translational step of comparing these proteomic changes to human liver diseases.

#### Liver tissue proteome analysis

A number of liver tissue proteome analyses have been reported in rodent models. Low *et al*. characterized the proteome of rat livers treated with thioacetamide (TAA). Using several time points, they proposed that TAA causes chronic liver injury leading to liver fibrosis through down-regulation of enzymes related to fatty acid β-oxidation, branched chain amino acids and methionine breakdown through depletion of succinyl-CoA and subsequent alterations in heme and iron metabolism [57]. Using carbon tetrachloride (CCl_4_) as a model for toxic liver injury in mice, and Abcb4-knockout mice as a model for sclerosing cholangitis to study differentially expressed proteins in liver tissue, Henkel *et al*. combined DIGE with MALDI-TOF MS and peptide mass fingerprint database search. They identified 20 differentially expressed genes in the CCl_4 _model and 8 genes in the Abcb4-knockout model compared to control mice [58]. A number of studies have characterized proteome changes after ethanol feeding in rats. Shepard *et al*. observed that many mitochondrial proteins are acetylated in livers of rats that are chronically ethanol-fed [59]. A study in ethanol-fed rats treated with S-adenosylmethionine (SAM) demonstrated that the proteins affected by ethanol and SAM treatments were chaperones, beta oxidation proteins, sulfur metabolism proteins and dehydrogenase enzymes involved in methionine, glycine and choline metabolism [60]. Kharbanda *et al*. treated ethanol-fed rats with betaine, which restores the metabolic ratio of liver S-adenosylmethionine to S-adenosylhomocysteine. Ethanol significantly reduced carbonic anhydrase-III protein levels, which can lead to decreased resistance against oxidative stress [61].

Rodent studies are also being used as models for fibrosis resolution after the chronic injury is removed. Liu *et al*. characterized liver tissue proteome changes during spontaneous recovery after TAA-induced micronodular cirrhosis in rats. Using 2-DE and MALDI-TOF, they identified the up-regulation of GST-P2 that peaked after two weeks of recovery [62].

Employing a cDNA microarray gene chip and 2D-DIGE with MALDI-TOF/MS-MS, Kirpich *et al*. found that glutathione S-transferases mu-1, pi-1 and selenium-binding protein 2 are decreased at both gene and protein levels in a mouse model of NAFLD [63].

Enoyl-coenzyme A hidratase, an enzyme that catalyzes the second step of mitochondrial fatty acid beta-oxidation, has been described by Zhang *et al*. to be down-regulated in rats with HFD-induced hepatic steatosis [64].

#### Hepatic stellate cell proteome analysis

Activation of HSCs is a key event in fibrogenesis and can be reproduced *in vitro *by prolonged culture on plastic or collagen. The changes in protein expression during activation can provide novel targets for antifibrotic therapy. However, differences between *in vitro *and *in vivo *HSC activation need to be taken into account. Kristensen *et al*. isolated hepatic stellate cells from rats and compared the proteome of quiescent cells with that of either culture-activated cells or *in vivo *activated cells from rats treated with CCl_4 _for eight weeks. Following 2-DE, proteins were digested by trypsin, and ESI-MS/MS was used to sequence the proteins that were differentially expressed. They detected 16 proteins showing differences based on the model of activation [65].

A study by Kawada *et al*. explored the proteome of *in vivo *(thioacetamide) and *in vitro *activated rat HSCs, describing a protein that was induced with HSC activation: stellate-cell activation-associated protein (STAP), which may act as an anti-fibrotic scavenger of peroxides during liver injury [66].

Deng *et al*. employed human hepatic stellate cells (LX2) treated and untreated with taurine and performed a combination of two-dimensional gel electrophoresis and ultra-performance liquid chromatography-electrospray ionization-tandem mass spectrometry (UPLC-ESI-MS/MS). They postulate a beneficial role of taurine in hepatic fibrosis, as they were able to detect an increased rate of stellate cell apoptosis after treatment [67].

#### Serum and urinary proteome analysis

Animal models could provide general serum markers that correlate with fibrosis stage and have the advantage of easier access to liver tissue at set time points for histological comparison. In thioacetamide (TAA) and bile-duct ligation treated rats, Xu *et al*. indentified and sequenced a 3.5 kDa histidine-rich glycoprotein, which had > 92% specificity and > 97% sensitivity for identifying liver cirrhosis. Previous studies have shown a beneficial effect of bone marrow cell (BMC) transplantation in CCl_4_-induced cirrhosis in mice, improving liver regeneration, function and fibrosis. Yokoyama *et al*. found six differentially expressed proteins in the serum by 2-DE, 48 hours after BMC transplantation, compared to non-transplanted mice. They suggested ApoA1 protein levels as a proteomic analysis of serum marker proteins in recipient mice with liver cirrhosis after bone marrow cell transplantation. marker for liver regeneration after BMC transplantation [68]. Serum taurocholic acid has also been recently proposed as a marker of early hepatic damage based on the Shimada *et al*. study. The identified elevated serum taurocholic acid concentration by SPE-MALDI-TOF MS in mice CCl_4 _induced liver injury [69].

A recent publication looked at urinary samples to find biomarkers for CCl_4_-induced liver fibrosis providing a potential alternative to serum samples for non-invasive diagnostics and follow-up in liver fibrosis (70).

### Chronic hepatitis C viral infection (HCV)

In patients with HCV, early knowledge of which patients have rapidly progressing fibrosis could be very beneficial in the decision-making process of whether to treat patients aggressively with antiviral therapy. A recent review by Walters and Katze explored the relationship among the gene expression profile of HCV patients, viral clearance and treatment response, shedding light on potential virus-host interactions, which may emerge as future therapeutic targets [71].

#### Staging of fibrosis

The liver proteome of HCV-patients can provide HCV-specific and general fibrotic pathways that could lead to novel therapeutic targets, or predict the natural disease progression or therapeutic response in the individual patient. Recent findings point towards alterations in fatty acid oxidation, oxidative phosphorylation and structural proteins in advanced fibrosis. An overview of high-throughput studies analyzing pathways and proteins involved in fibrosis due to HCV is represented in Table 1.

**Table 1:** High-throughput studies in HCV patients analyzing differential gene expression based on fibrosis stage

1^st ^author	Year	# Patients	# Controls	Specimen	Methods	# Differentially expressed genes	Main pathways involved	Most differentially regulated genes	⇑/⇓	Reference
Huang	2006	121	312	Blood	SNP microarray	1,609	N/A	DEAD-box popypeptide 5 (DDX5) SNP Carnitine palitoyltransferase 1A (CPT1A) SNP	⇑ ⇑	81
Diamond	2007	22	4	Liver tissue	LC-ESI-FTICR MS/MS	210	Amino acid, carbohydrate and lipid metabolism Oxidative phosphorylation Oxidative stress	N/A	N/A N/A N/A	72
White	2007	23	21	Serum	2-DE/LC-Q-TOF MS/MS	7	N/A	Alpha-2-macroblobulin Haptoglobin Fragment of albumin Serum retinol binding protein (SRBP) Complement 4 Apolipoprotein A-I (Apo A1) Apolipoprotein A-IV (Apo A4)	⇑ ⇑ ⇑ ⇓ ⇓ ⇓ ⇓	74
Gangadharan	2007	11	4	Serum	2-DE-Q-TOF/CapLC MS/MS	83	Plasmin-associated Hepatic synthetic function HGF-releated Lipid metabolism Immune system related	Alpha-2-macroblobulin Inter-alpha-trypsin inhibitor heavy chain 4 Albumin Transthyretin Complement C3 and C4 Factor H-related protein 1 Alpha-1 antichymotrypsin Haptoglobin Apolipoprotein L-I Beta 2 glycoprotein CD5 antigen like IgA1+IgG2 heavy chain and Ig light chain regions	⇑ ⇓ ⇓ ⇓ ⇓ ⇓ ⇓ ⇓ ⇓ ⇓ ⇑ ⇓ ⇓ ⇑ ⇑	76
Mölleken	2009	7	7	microdissections	2-DE/LC-ESI-Ion trap MS/MS	35	Cell structure	Tropomyosin alpha Tropomyosin beta Microfibtil-associated glycoprotein 4 Calpoin 1 Transgelin Tropomyosin alpha 4 Actin alpha 1 Vimentin	⇑ ⇑ ⇑ ⇑ ⇑ ⇑ ⇑ ⇑	73

Diamond *et al*. have described a pattern of 210 proteins that correlate with fibrosis stage in 1,641 HCV-infected patients. They were able to cluster the patients in fibrosis stages 3 to 4 versus stages 1 to 2 using these protein expression profiles. Functional analysis indicated reduced expression of genes involved in fatty acid oxidation and oxidative phosphorylation in advanced fibrosis [72]. Several cell structure-associated proteins were uncovered in the cirrhotic septa of seven HCV patients (METAVIR stage F4) when Mölleken *et al*. compared the proteomic profiles of microdissected cirrhotic septa versus parenchymal liver cells using 2-DE and LC with ESI-MS/MS, in the search for new serum biomarkers for fibrosis. MFAP-4 could predict the presence of fibrosis with high accuracy in both HCV- and alcohol-induced fibrosis, but diagnostic accuracy decreased significantly for the discrimination between fibrosis stages 2 to 4 [73].

Regarding serum markers, none of these serum markers can adequately distinguish intermediate fibrosis stages, even though the progression from mild to intermediate fibrosis is an important predictor of further fibrosis progression. White *et al*. reported three up-regulated proteins (α2-macroglobulin, haptoglobin, albumin) and four down-regulated proteins (complement C-4, serum retinol binding protein, apolipoprotein A1, and two isoforms of apolipoprotein A4) in the serum of HCV patients with advanced fibrosis (METAVIR stage F3/4, n = 23) versus no or mild fibrosis (METAVIR stage F0/1, n = 21) [74]. Three of these proteins are already being used in the FibroTest [75]. This study was limited by a small sample size, and the four new potential biomarkers could not be validated in a larger patient group. Gangadharan *et al*. found a decrease in several proteins, including apolipoprotein L1, prealbumin, albumin, haptoglobin and complement components, whereas other proteins, such as CD5 antigen-like (CD5L), 2-macroglobulin and immunoglobulin components, were induced in fibrosis when they studied the serum proteome of HCV patients with varying levels of fibrosis and healthy controls, using 2-DE and LC with Q-TOF MS/MS [76].

A novel set of four cDNA peripheral blood mononuclear cell markers (a2-macroglobulin, haptoglobin, mitogen-activated protein kinase kinase 3, and alanine aminopeptidase N) accurately predicted the stage of liver fibrosis in HIV/HCV co-infected patients with an area under the receiver operating curve (AUC ROC) of 0.852 [77].

L Yang *et al*. recently analyzed 24 liver samples from patients with chronic HCV by DIGE and Isobaric tags for relative and absolute quantitation (iTRAQ) and identified 2 proteins as biomarker candidates for predicting hepatic fibrosis: complement C4-A and inter-alpha-trypsin inhibitor heavy chain H4 [78,79].

### Novel biomarker candidates to predict hepatic fibrosis in hepatitis c identified by serum proteomics

Fibrosis progression has also been related to galectin-3-binding protein (G3BP). Cheung *et al*. found increased levels in patients with hepatitis C-related cirrhosis when compared with mild and moderate fibrosis in 76 serum and 20 tissue human samples [80].

#### Prediction of fibrosis progression

Prediction of fibrosis progression is crucial for adequate patient selection in clinical trials, and, thus, Huang *et al*. studied the genome of 1,468 patients with chronic hepatitis C infection and known fibrosis stage. They developed a Cirrhosis Risk Score (CRS) consisting of SNPs in seven genes, which could predict fibrosis progression significantly better than clinical parameters. Four SNPs were in known genes: antizyme-inhibitor-1, toll-like receptor 4, transient receptor potential cation channel, subfamily M, member 5 and aquaporin 2 [81]. This initial study was independently validated two additional studies [82,83], and is especially exciting, as it is the first evidence that host genetics significantly influence fibrosis progression and, thereby, provides a means for selecting patients that will benefit most from aggressive antiviral or antifibrotic treatment.

At the proteomic level, when plasma from cirrhotic HCV patients is analyzed by SDS-PAGE and MS, both chains of the complement C3 and C4 show a significant decrease in comparison with samples from healthy patients [84,85].

#### Prediction of treatment response

Transcriptomic and proteomic studies point to a crucial role of interferon signaling pathways in the prediction of treatment response, where high pre-treatment interferon signaling is associated with poor virological response. Recent genomic studies have identified SNPs in several interferon-related genes, especially interleukin 28b, that are powerful predictors of virologic response indicating that genomics can be a powerful tool in the prediction of treatment response.

A recent publication by Asselah *et al*. demonstrated that liver gene expression can predict outcome of patients with chronic hepatitis C infection after treatment with pegylated interferon and ribavirin. They studied cDNA of non-responders (NR), and SVR using RT-PCR for 59 genes. Three genes were significantly induced in NRs versus SVRs: interferon alpha-inducible protein 3, interferon alpha-inducible protein 27 and interferon alpha-inducible protein 2. Supervised class prediction analysis identified a two-gene (interferon-alpha-inducible protein 27 and CXC chemokine ligand 9) signature, with a predictive accuracy of 100% in NRs and of 70% in SVRs. Although no high-throughput analysis was performed, this paper provides a basis for further exploring the use of genomic and proteomic information to predict individual treatment response, and may be useful for selecting those patients who will benefit from treatment [86].

Chen *et al*. analyzed liver tissue using cDNA microarray to identify a gene signature that can predict treatment outcome. A key finding was that interferon stimulated gene 15 (ISG15) protein induction was more pronounced in hepatocytes in non-responders, but in Kupffer cells in responders [75]. In a liver tissue microarray study by Hayashida *et al*., a different set of genes was found to predict treatment response in patients treated with interferon alone, or interferon/ribavirin co-treatment. In the interferon monotherapy patients, main predictors were genes involved in the interferon signaling pathway, lipid metabolism, complement and oxidation/reduction. In the combined treatment group, genes, including *cylophylin A*, and multidrug resistance protein-hepatic gene expression during treatment with peginterferon and ribavirin emerged, identifying molecular pathways for treatment response. 1 (P-gp) [87]. Feld *et al*. discovered that low baseline liver tissue cDNA levels of interferon-stimulated genes (ISGs) predict rapid treatment response with quick induction of ISG levels, whereas high baseline ISG levels do not increase much further and predict slow treatment response, together with up-regulation of interferon inhibitory pathways [88].

As described above, recent genome-wide association studies (GWAS) have identified SNPs in the interleukin 28b (IL28B, coding for interferon-γ-3) genomic region that are associated with viral response to the current treatment of HCV, pegylated-interferon-alpha (PEG-IFNα) and ribavirin. GWAS by Suppiah *et al*. led to the discovery of a SNP near interleukin 28b (IL28B) that is associated with sustained viral response (SVR) in genotype 1 HCV patients treated with PEG-IFNα and ribavirin, with an odds ratio (OR) for the G-allele of rs8099817 of 1.64 for heterozygous carriers and 2.39 for homozygous carriers compared to non-carriers [89]. Other SNPs that were associated with SVR included Caspase 1, a known activator of interleukin 1, and interleukin 21 receptor. A simultaneous report was published by Tanaka *et al*., identifying SNPs in IL28B in a Japanese genotype 1 HCV-patient cohort as a predictor of virologic response to PEG-IFNα and ribavirin. Initial GWAS identified rs2980275 and rs8099917 SNPs as predictors of non-response and SVR. They continued to seek SNPs in the IL28A/B region and uncovered rs8105790, rs11881222, rs8103142, rs28416813, rs4803219, rs8099917 and rs7248668 as associated with virologic response [90]. Identification of those HCV-patients who are likely to respond to therapy could lead to a shortened duration of therapy in patients with a favorable SNP genotype. Although these studies did not examine fibrogenic end-points, they are a major breakthrough in HCV treatment response research, and are excellent examples of how GWAS can identify genes and SNPs that are related to therapeutic responses.

Non-responders generally have high expression of interferon-stimulated genes (ISGs) pre-treatment that cannot be further induced by PEG-IFNα, whereas RVR patients have strong induction of ISGs on treatment. Refractory high endogenous interferon signaling in non-responders may interfere with PEG-IFNα treatment, especially in genotype 1 and 4 patients. Whether inhibition of endogenous interferon signaling could improve therapeutic response is subject to further study [91,92].

Although further validation is needed, Devitt E *et al*. using SELDI-TOF MS analyzed serum from 25 HCV infected patients during the initial 24 h of treatment with pegylated interferon α-2b and ribavirin and identified 16 differential peaks able to distinguish responders from nonresponders [93].

### Chronic hepatitis B viral infection (HBV)

Approximately 400 million patients worldwide are chronically infected with hepatitis B virus (CHB). Hepatitis B virus infection causes chronic hepatitis in about 10% of infected patients, and increases the risk of developing HCC about 100-fold versus in the uninfected population. An excellent review on the role of genomics, transcriptomics and proteomics in chronic HBV-associated liver disease, recently published by Sun *et al*., described determinants of susceptibility to persistent HBV infection, disease progression and HCC development; their review also describes the use of these technologies to understand the pathogenic mechanism of HBV and to identify biomarkers that could aid in early detection of HCC [94].

Recent high-throughput proteomic studies have identified protein patterns that correlate with fibrosis stage in HBV patients. Zhang *et al*. described a novel approach to evaluate the protein expression profile of plasma membrane and analyzed human liver samples from HBV patients in different fibrosis stages. Using 2-DE, IPGphor isoelectronic focusing system and Bio-Rad Protein II electrophoresis to the investigators identified positive correlation between fibrosis grade and annexin A2 levels [95]. Lu *et al*. analyzed plasma of 7 healthy controls and 27 HBV patients with different stages of fibrosis by 2-DIGE and identified the up-regulation of peroxiredoxin 2 as a potential biomarker of HBV related liver fibrosis [96]. Although not liver-specific, plasma gelsolin protein has been pointed out as a potential predictor of HBV progression. C Marrocco *et al*. found by 2-DE a repressed expression of the protein in plasma of eight cirrhotic HBV patients, compared to eight chronic HBV infected patients [97]. Mohamadkhani *et al*. performed a 2DE and mass spectrometry analysis in patients with either chronic hepatitis B or HBV-related cirrhosis and healthy controls. Their data suggest that progression of HBV-related liver injury is associated with a down-regulation of apolipoprotein A1 and an increase in myeloperoxidase levels [98]. However, these results still need to be validated, and their ability to distinguish intermediate fibrosis stages is not clear.

Poon *et al*. using SELDI ProteinChip arrays and the Significance Analysis of Microarray (SAM) algorithm generated a model able to predict fibrosis (Ishak score ≥ 3) and cirrhosis (Ishak score ≥ 5) with an accuracy of > 90% [99]. Liver histology (METAVIR score) of HBV-patients was compared with three sets of serum markers by Myers *et al*.: aminotransferases (ALT, AST), Fibrotest (α_2_-macroglobulin, apolipoprotein A1, haptoglobin, gamma-glutamyl-transpeptidase (GGT) and total bilirubin) and Actitest (ALT). Aminotransferases and Actitest were good predictors of activity (stage A2-F3) and Fibrotest accurately predicted stage F2-F4 fibrosis, especially in the ranges of ≤ 0.20 or > 0.80. Limiting liver biopsy to patients with intermediate Fibrotest scores could prevent biopsies in about half of the patients, without affecting accuracy [100].

Wang *et al*. performed an *in vitro *study to investigate proteome differences between stably HBV transfected HepG2.2.15 versus wild type HepG2 cells by 2-DE followed by LC-ESI-MS/MS. Furthermore; they compared HepG2.2.15 cells with and without interferon-alpha (IFN-α) treatment. Proteins that were differentially expressed in HepG2.2.15 versus HepG2 cells could be classified as being involved in, for example, cytoskeletal matrix, heat shock stress, signal transduction and protease/proteasome components. IFN-α treatment induced expression of interferon-stimulated gene 15 (ISG15) and prohibitin, among others, providing potential pathways that could lead to tumor suppression and defense against viral infection. Although these results have not been validated *in vivo*, they nevertheless establish that proteomics and cell culture can be a powerful combination to uncover differences in the proteome, and can be useful for evaluating potential treatments [101].

### Alcoholic liver disease (ALD)

Several genomic and proteomic studies have been published on patients with alcoholic liver disease. However, the majority of studies focus on either diagnostics or genes/proteins that differentiate alcoholic hepatitis from alcoholic steatosis, not in predicting progression towards fibrosis and cirrhosis. Nevertheless, alcoholic steatohepatitis is clearly a precursor for developing fibrosis. For example, 586 genes were differentially expressed between alcoholic hepatitis and alcoholic steatosis, and 211 genes were differentially expressed between alcoholic hepatitis and non-disease controls when Seth *et al*. compared liver tissue RNA from eight patients with alcoholic hepatitis, nine with alcoholic steatosis, and seven non-disease controls using microarray analysis. Induced genes included pathways controlling cell adhesion, immune response, oncogenesis, signal transduction and embryogenesis. The 111 down-regulated genes included pathways for protein synthesis, cell growth and maintenance, transcription, signal transduction and transport [102].

### Non-alcoholic fatty liver disease (NAFLD) and non-alcoholic steatohepatitis (NASH)

The prevalence of NAFLD and NASH is on the rise, which makes these increasingly important causes of liver fibrosis, especially in countries with a Western diet containing a high fat and/or carbohydrate content. For NAFLD and NASH there is also a lack of genomic/proteomic studies specifically examining fibrogenesis and fibrosis progression, much like in alcoholic liver disease. Most genomic/proteomic studies focus on distinguishing NASH from NAFLD, and not on progression to liver fibrosis and cirrhosis. The ability to distinguish NASH from NAFLD is very relevant, however, as NASH clearly predisposes to fibrosis. Therefore, we reviewed recent publications on this topic. However, studies directed at identifying proteins related to the speed of fibrosis progression in NASH could further select patients who could benefit from treatment, improve understanding of fibrosis pathogenesis in fatty liver, and identify targets for treatment of NASH-related fibrosis.

#### Staging of steatosis

NASH is distinguished from NAFLD by an inflammatory component. Therefore, not surprisingly, genes that are up-regulated in NASH versus NAFLD have strong relationships with inflammatory and immune pathways. Complementary DNA microarray analysis on nine liver tissue samples from steatotic livers and nine normal livers unearthed 110 differentially expressed genes, many related to mitochondrial respiratory and mitochondrial metabolic pathways. Genes belonging to the interleukin-1 receptor family and transforming growth factor-beta were induced in steatotic livers, pointing to a deranged inflammatory pathway, even in early development of steatosis. Thirty-four genes with significant differential expression were identified in patients with NASH, when compared with non-obese controls in a study by Younossi *et al*., using cDNA microarray analysis on liver biopsies taken during bariatric surgery to find differential expression patterns between NASH (n = 29), NAFLD (n = 12), obese non-steatotic (n = 7), and non-obese controls (n = 6) [103]. Nineteen of these genes showed no significant expression differences in obese versus non-obese controls, suggesting a stronger association of these genes to NASH. These genes were related to lipid metabolism, extracellular matrix remodeling, liver regeneration, apoptosis and detoxification. In another study, this group used parallel liver tissue gene expression and serum proteome analysis in a group of 98 morbidly obese patients. Twenty-seven patients had developed NASH, 52 patients had steatosis with nonspecific inflammation, 12 patients had steatosis alone and 7 patients had normal histology on biopsy. Using SELDI-TOF analysis on serum samples, this group found several protein peaks associated with different stages of NAFLD/NASH. However, except for fibrinogen γ, the corresponding proteins were not identified. On the mRNA level, insulin-like growth factor binding protein 1 (IGFBP1) and fatty acid CoA ligase 4 (FACL4) were differentially expressed between NASH and steatotic patients.

A recent study of the serum proteome by Bell *et al*. led to the discovery of 55 proteins that changed significantly between simple steatosis and NASH with stage F3/F4 fibrosis (advanced bridging fibrosis/cirrhosis), and 15 proteins that changed between early NASH and NASH with stage F3/F4 fibrosis. Most of these proteins are involved in the immune response, coagulation, cellular and extracellular matrix and blood carrier proteins [104]. These proteins shed light on the pathogenesis of NAFLD/NASH and could be explored as candidates for biomarkers of advancing disease. Ulukaya *et al*. were able to use serum MALDI-TOF MS peaks to accurately predict NAFLD versus controls, but could not distinguish between simple steatosis and NASH. They included 80 patients with NAFLD, and 19 controls [105]. Histologic classification of the NAFLD patients showed definite NASH in 48 patients, borderline NASH in 22 patients and 10 patients with simple steatosis. Using reverse phase protein microarray technology, Calvert *et al*. revealed deranged insulin signaling in NAFLD patients when they investigated omental adipose tissue from 99 obese patients. Liver biopsies were classified as follows: (1) no fatty liver disease present, (2) simple steatosis, (3) steatosis with nonspecific inflammation, or (4) NASH. Categories 2 to 4 were clustered as NAFLD. Protein kinase A (PKA) and AKT/mTOR deregulation and caspase 9 activation were good predictors of NASH on liver histology compared to non-progressive forms of NAFLD [106]. For more information on genomic and proteomic analysis of NAFLD and NASH, see a recent review by Wilfred de Alwis and Day [107].

#### Fibrosis progression in steatotic patients

Very few publications report changes in gene expression that correlate with fibrosis stage in NAFLD/NASH patients. So far, only apolipoproteins, CD5 antigen-like (CD5L) and lumican have been identified. Gray *et al*. applied 2-DE and MALDI-TOF to analyze differences in serum proteome profile between patients with pre-cirrhotic NAFLD (n = 5), cirrhotic NAFLD (n = 5) and cirrhotic NAFLD with HCC (n = 5). Four out of five spots that identified at least one of the three groups were identified as apolipoprotein isoforms. The fifth spot, identified as CD5L, was induced in cirrhotic patients and even more in patients with HCC. ELISA analysis of CD5L in a larger patient group showed a poor AUROC score for distinguishing patients with HCC from those without HCC. However, as a cirrhosis biomarker, ROC analysis showed an accuracy of 72%. CD5L could potentially be used in combination with other biomarkers for cirrhosis [108]. Charlton *et al*. identified lumican, a 40 kDa keratin sulfate proteoglycan, as significantly increased in liver tissue from patients with mild NASH (NASH with F0/F1 fibrosis) versus simple steatosis, and in patients with progressive NASH (NASH with F2-F4 fibrosis) versus mild NASH in obese patients, when analyzed with LC-Q-TOF MS/MS. FABP-1 was increased in simple steatosis patients compared to non-steatotic patients, but was paradoxically decreased in NASH patients, which suggests an impaired detergent effect against free fatty acids in progressive NAFLD patients [109].

### Autoimmune liver diseases

Primary biliary cirrhosis (PBC), as well as related autoimmune disorders, is more prevalent in certain families and thus seems to have a complex genetic component, with many factors probably adding to the risk of developing PBC. Some of these genes associated with PBC are major histocompatibility complex genes and common autoimmune genes [110]. Shackel *et al*. discovered that PBC liver tissue had increased expression of Th1 and Th2 type proteins as well as connective tissue growth factor and TGF-β3 when they analyzed their cDNA microarray from liver tissue from patients with PBC (n = 4), PSC (n = 4) and non-disease controls (n = 8). Moreover, Wnt and Notch signaling pathways were induced. PSC patient samples had increased expression of apoptosis associated molecules [111]. Serum samples from 44 PBC patients and 75 controls, analyzed using WCX magnetic beads and MALDI-TOF-MS, provided 69 potential protein biomarkers. Li *et al*. used these to construct a diagnostic model for PBC using the *m/z *peaks of protein biomarkers 3445, 4260, 8133, and 16290, which showed a sensitivity of 92.9% and a specificity of 82.4% [112].

Tahiri *et al*. noticed an induction of six known potential plasma membrane expressions: liver arginase, cytokeratins 8/18, heatshock proteins (HSP) 60/70/90, and valocin-containing protein (VCP) in autoimmune hepatitis when they investigated serum samples from 65 autoimmune hepatitis type 1 patients and 90 controls consisting of healthy blood donors (n = 40) and patients with systemic diseases (n = 20) or other liver diseases (n = 30). They proposed that these proteins could be targets for auto-antibodies in autoimmune hepatitis [113]. Using protein microarray, Song *et al*. analyzed serum samples from patients with autoimmune hepatitis (n = 44), PBC (n = 50), HCV (n = 41), HBV (n = 43), systemic lupus erythematosus (SLE) (n = 11), primary Sjögren syndrome (n = 11), rheumatoid arthritis (n = 2) and 50 healthy controls. Initially they found 11 differentially expressed genes between autoimmune hepatitis and other samples. They then produced an autoimmune hepatitis-specific protein chip, and found three new antigens, ribosomal protein S20 (RPS20), Alba-like, and dUTPase, that could be used as highly specific biomarkers for autoimmune hepatitis, and validated this with ELISA [114]. Bowlus *et al*. have recently analyzed by *in situ *MALDIMS (matrix-assisted laser desorption/ionization mass spectrometry) in PBC (n = 29), PSC (n = 11), AIH (n = 7) and healthy controls (n = 10) and found a promising pattern of protein expression in bile ducts, inflammatory infiltrates and hepatocytes that may represent a feasible way to identify novel targets in these diseases [115]. Song *et al*. created a protein microarray containing 5,011 recombinant human liver proteins and were able to identify three new antigens, RPS20, Alba-like and dUTPase, as highly autoimmune hepatitis-specific biomarkers [114].

Although these results are promising, the number of high-throughput genomic and proteomic studies in autoimmune liver diseases is still very limited.

### Hepatocellular carcinoma

While beyond the scope of this review, there have been significant advances in early diagnosis, treatment and pathway analysis of HCC and hepatocarcinogenesis through genome, transcriptome and proteome high-throughput analysis, especially in the setting of liver cirrhosis. For more information on this topic we refer to recent reviews [116-118].

## General conclusions and future directions

In the last decade, genomic and proteomic hypothesis-free studies have become increasingly common. Since liver fibrosis often takes many years to develop, and not every patient will become symptomatic, a method to select patients who will benefit from aggressive treatment in an early phase would be very beneficial. Therefore, there is a demand for biomarkers that can provide a prognostic indication for fibrosis, either in the natural course of chronic liver disease, or as a predictor of treatment response. Liver biopsy is currently the gold standard for follow-up in chronic liver disease, but it can have serious complications and suffers from sampling error and inter-observer variation. This drives the search for non-invasive direct or indirect biomarkers that correlate accurately with fibrosis stage.

Prospective predictive proteomic and genomic studies are difficult in liver disease, as follow-up is long, and biological samples can degrade over time. Thus far, many tests based on serum biomarkers correlate fairly well with the fibrosis stage when advanced fibrosis (METAVIR F3-4) is compared to mild or no fibrosis (F0 to 2), but lack resolution in the intermediate stages. Especially, these intermediate stages (F1 to 2) could be helpful in the early detection of progressive fibrosis and guide the hepatologist in treatment decisions. So far, genomic and proteomic studies for fibrosis staging have been mostly limited to viral hepatitis, with other diseases like alcoholic liver disease and NASH yet to be explored. Studying the fibrotic genome/proteome in these diseases will shed light on common and distinct pathological pathways leading to liver fibrosis.

Genomic and proteomic studies that have identified genes and proteins that correlate with fibrosis levels have yielded heterogeneous findings, most likely due to variations in sample preparations and patient populations/characteristics. The lack of standardized sample preparation protocols hinders reproducibility of protein spectra. A few gene and protein groups seem to correlate with fibrosis more consistently, mostly belonging to glutathione, oxidative stress, inflammatory and immune response pathways. For most of the identified proteins, adequate follow-up has been lacking so far, leaving scientists with lots of potential biomarker candidates still in need of validation.

Future high-throughput research will continue uncovering genes and proteins that can elucidate common and specific-disease pathways that lead to liver fibrosis. Standardization of sample preparation protocols should increase reproducibility of these studies, and will make it easier to select candidate genes and proteins that are suitable for validation as biomarkers for either fibrosis staging, or as predictive biomarkers for disease outcome and/or treatment response.

## Abbrevations

2-DE/2D-PAGE: 2D-polyacrylamide gel electrophoresis; AIH: autoimmune hepatitis; ALD: alcoholic liver disease; BMC: bone marrow cell; CCl_4: _carbon tetrachloride; CRS: Cirrhosis Risk Score; DIGE: gel electrophoresis; ESI: electrospray ionization; FACL4: fatty acid CoA ligase 4; FT-ICR: Fourier transform-ion cyclotron resonance; GWAS: genome-wide association studies; HCC: hepatocellular carcinoma; HCV: chronic hepatitis c viral infection; HSCs: hepatic stellate cells; IGFBP1: insulin-like growth factor binding protein 1; IMAC: immobilized metal affinity capture; ISGs: interferon-stimulated genes; iTRAQ: isobaric tags for relative and absolute quantitation; LC: liquid chromatography; MALDI: matrix-assisted laser desorption/ionization; MALDIMS: matrix-assisted laser desorption/ionization mass spectrometry; MS: mass spectrometry; m/z: mass-to-charge; NAFLD: non-alcoholic fatty liver disease; NASH: non-alcoholic steatohepatitis; OR: odds ratio; PBC: primary billiary cirrhosis; PCR: polymerase chain reaction; PKA: protein kinase A; Q-Q-TOF: second quadruple time-of-flight; Q-TOF: quadruple time-of-flight; RT-qPCR: reverse transcription quantitative PCR; SAGE: serial analysis of gene expression; SAM: significance analysis of microarray; SAX: strong anion exchange; SELDI: surface-enhanced laser desorption/ionization; SNPs: single nucleotide polymorphisms; STAP: stellate-cell activation-associated protein; SVR: sustained viral response; TAA: thioacetamide; TIMP-1: tissue inhibitor of metalloproteinases-1; TOF: time-of-flight; UPLC-ESI: ultra-performance liquid chromatography-electrospray ionization-tandem; WCX: weak cation exchange

## Competing interests

The authors declare that they have no competing interests.

## Authors' contributions

RH participated in the design of the study, carried out the bibliographic search, drafted the manuscript and created the tables. VHG participated in the design of the study, the bibliographic search and the manuscript drafting. SF conceived of the study, participated in its design and coordination, and helped to draft the manuscript. All authors read and approved the final manuscript.
